# Early T Stage Is Associated With Poor Prognosis in Patients With Metastatic Liver Colorectal Cancer

**DOI:** 10.3389/fonc.2020.00716

**Published:** 2020-06-18

**Authors:** Lunpo Wu, Jianfei Fu, Yi Chen, Liangjing Wang, Shu Zheng

**Affiliations:** ^1^Department of Gastroenterology, Second Affiliated Hospital of Zhejiang University School of Medicine, Hangzhou, China; ^2^Institution of Gastroenterology, Zhejiang University, Hangzhou, China; ^3^Department of Medical Oncology, Jinhua Hospital, Zhejiang University School of Medicine, Jinhua, China; ^4^Cancer Institute (Key Laboratory of Cancer Prevention and Intervention, Chinese National Ministry of Education and Key Laboratory of Molecular Biology in Medical Sciences), Second Affiliated Hospital, School of Medicine, Zhejiang University, Hangzhou, China

**Keywords:** metastatic liver colorectal cancer (CRLM), survival, early T stage, survival, nomogram

## Abstract

Clinically, a considerable portion of patients with early T stage who were supposed to have a low distant metastatic probability were diagnosed with metastatic liver colorectal cancer (CRLM). Our study aims to evaluate the prognostic value of the T stage for metastatic patients and establish a convenient individual assessment model for clinicians to explore preoperative predictors. The mRNA profiles of colorectal tumors (*N* = 19) were obtained by microarray at our clinical center. A total of 5,618 patients with CRLM from 2010 to 2015 were enrolled for the Surveillance, Epidemiology, and End Results (SEER) database. The cDNA microarray analyses showed that gene expression pattern in the T2N0M1 subgroup was significantly different from the T3/4N0M0 subgroup. In the survival analysis, metastatic patients with T1 stage surprisingly had much poorer prognosis than those with T3/T4 stage. Specifically, metastatic patients with early T stage were observed with higher frequency occurring at the rectum, better differentiation, less metastases in the lymph nodes, and a higher CEA level. Further survival analysis indicated that early T classification was an independent prognostic factor for a poor survival. When the lymph node (N) status was taken into consideration, patients with T1/2N+ had better survival than T1/2N0 patients. A clinical nomogram was constructed based on preoperative factors. The calibration curves showed a good concordance between nomogram prediction and actual observation. In conclusion, CRLM with early T stage had a different biological background. The prognosis of patients at T1/2M1 was poorer than at T3/4M1. More attention should be paid to the surveillance of high-risk factors and the screening of early T stage.

## Introduction

With the launch of mass screening programs for colorectal cancer (CRC), there is an increasing trend of patients who were diagnosed at an early stage. According to newly released data from China (1), ~45.5% of diagnosed patients from screening were at an early stage, while 54.5% were metastatic patients. Approximately 25% of colorectal cancer cases present with synchronous metastatic disease (2, 3). Among them there is a subgroup of patients who were supposed to have relatively good prognosis, progressing dramatically during the follow-up period. T stage is often considered as a detailed and credible category of the depth of tumor invasion, and early T stage is always supposed to have a low disposition of distant spreading. Unexpectedly, a considerable portion of patients at early T stages who were supposed to have a low distant metastatic probability were diagnosed with metastatic liver colorectal cancer (CRLM).

Among untreated metastasis patients, the survival time is usually less than 12 months (4), while a resection with curative intent could render a dramatically improved 5-year survival rate (5, 6). However, there are still a substantial number of patients amenable to radical resection who will subsequently undergo recurrence or even decease shortly after surgical resection (7–9). These patients pose a significant challenge, and they might just not be suitable candidates for immediate metastasectomy strategy.

There are around 10% of patients having poor survivals despite having been diagnosed as “early” stage (10). It is noteworthy that even with aggressive treatment, a number of metastatic patients still have extremely poor prognosis. Therefore, it is necessary to point out whether there is a special but vicious tumor whose grade malignancy is high. The current American Joint Committee on Cancer (AJCC) staging system fails to characterize the T stage in metastatic CRC patients and limited reports have assessed its “real” effects on prognosis. Therefore, we aim to identify this group of patients from both biological and clinical aspects. Our study presents the “real” survival of CRLM patients with early T stage and further explores preoperative predictions to establish a convenient individual assessment model for clinicians to speculate patients, with poor prognosis.

## Materials and Methods

### Data Collection

The Surveillance, Epidemiology, and End Results (SEER) program is an authoritative American Cancer Information Database which is sponsored by the National Cancer Institution with the aim of collecting information about cancer incidence and survival (http://seer.cancer.gov/). SEER collects cancer incidence data from population-based cancer registries covering ~34.6 percent of the U.S. population. The SEER registries collect data on patient demographics, primary tumor site, tumor morphology, stage at diagnosis, and first course of treatment, and they follow up with patients for vital status. We obtained permission to access the data via SEER. Stat software (SEER^*^Stat 8.3.6). The patients diagnosed after 2015 were excluded to ensure an adequate follow-up duration.

### Patient Enrollment

The following specific inclusion criteria were considered: (1) Year of diagnosis was from 2010 to 2015. (2) Site record ICD-O-3 was limited to the colon and rectum. (3) Histological type ICD-O-3 was limited to 8140, 8480, 8481, and 8490 (adenocarcinoma, mucinous adenocarcinoma and signet ring cell cancer). (4) The stage was confirmed as IV according to the 7th American Joint Committee on Cancer (AJCC) system, including IVa and IVb. The following exclusion criteria were considered: (1) Patient whose primary tumor or regional lymph nodes were not removed. (2) Patient lacking documentation on race, age at diagnosis and differential grade. (3) Patient younger than 20 years old or older than 80 years old. (4) Patient with multiple primary tumors. (5) Patient who survived less than one month was excluded because they may die of surgical complications or quickly progress after palliative resection.

### Extraction of Total RNA

Freshly frozen tissues of primary colorectal tumors in 19 patients with resectable CRLM (2 patients with T2N0M1 and 17 patients with T3/4N0M0) were obtained from the Second Affiliated Hospital of Zhejiang University School of Medicine. All tissue samples were collected, immediately snap-frozen in liquid nitrogen, and stored at −80°C until RNA extraction. Written informed consent from each patient was obtained according to the institutional regulations. Total RNA isolation was performed with TRIzol (Invitrogen, Carlsbad, CA) according to the instructions of the manufacturer. The RNA concentration was determined using the NanoDrop-1000 Spectrophotometer (NanoDrop Technologies, Wilmington,DE). The 2100 Bioanalyzer (Agilent Technologies, Santa Clara, CA) was used to assess the integrity of the RNA. All RNA samples used in this study having a 260/280 ratio above 1.8 and an RNA integrity number greater than 5.0. Written informed consent from each patient was obtained according to the institutional regulations. The study was approved by the ethics committee of Second Affiliated Hospital of Zhejiang University School of Medicine.

### cDNA Microarray

Cyanine-3 (Cy3)-labeled cRNA was prepared from 0.5 mg total RNA using the One-Color Low RNA Input Linear Amplification PLUS kit (Agilent), followed by RNeasy column purification (QIAGEN, Valencia, CA). A total of 1.65 mg of Cy3-labeled cDNA (specific activity >6.0 pmol) was fragmented and hybridized to Agilent 4_44K Whole Human Genome Oligo Microarrays (G2600D) using the Gene Expression Hybridization Kit (Agilent). After hybridization, the microarrays were washed with the Gene Expression Wash Buffer Kit (Agilent) and scanned with Agilent's Feature Extraction 9.1 software with default parameters. The microarray data have been submitted in NCBI's Gene Expression Omnibus with the series accession number GSE146480.

### Analysis of mRNA Profiles

The statistical analysis of microarray data was performed with the GeneSpring GX Analysis Software v11.5.1 (Agilent). Raw data were preprocessed by log 2 transformation, and normalization between all arrays was performed using the 95th percentile method. Analyses where 100% of the samples in any condition had values were included. Differentially expressed genes were identified if the fold-change ≥5.0. Heat-map and clustering were performed in R software (version 3.0.3). Hierarchical clustering with average linkage using the Pearson correlation as a distance metric was applied to cluster the samples according to their mRNA expression levels.

### Statistical Analysis

Tumor location was classified as rectum and colon [including rectosigmoid junction, sigmoid colon, descending colon, splenic flexure, transverse colon, hepatic flexure, ascending colon, large intestine, none of specific (NOS)]. Race was divided into white, black and others. Age was classified into young (≤ 60 years old) and old (>60 years old) groups. Marital status was divided into married, divorced, single, and unknown. All cases were regrouped according to the 7th AJCC TNM staging system. The reference value of carcinoembryonic antigen (CEA) is defined as <2.5 ng/ml for non-smokers and <5 ng/ml for smokers. Subsequently, CEA was classified into negative, positive and unknown groups. The metastatic site was divided into liver, lung, bone, brain, and others.

Overall survival (OS) was calculated from the date of diagnosis to the date of all cause death. Survival curves were generated using Kaplan-Meier methods, and the log-rank test was performed to evaluate the survival differences among groups. Adjusted hazard ratios (HRs) along with 95% confidence intervals (CIs) were calculated using the Cox proportional hazards regression model. When the two-sided *P* value was < 0.05, the difference was considered statistically significant. A nomogram was formulated based on the results of the Cox proportional hazard model and by using the package of *rms* in R software (http://www.r-project.org/). For inclusion into the final nomogram model, effect of the continuous variable, age was explored using restricted cubic splines with five knots which made a satisfied sensitivity. The bootstrap validation method (bootstraps with 1,000 resample) was used to estimate the bias-corrected or overfitting-corrected predictive accuracy of the model, which is presented by concordance index (C-index). Calibration curves, which plot the average Kaplan-Meier estimate against the corresponding nomogram model for 1−, 3−, or 5− year predicted OS, are provided to evaluate the performance of the nomogram. When the two-sided *P* value was < 0.05, the difference was considered statistically significant. Analyses were performed using statistical software STATA/SE 13.0 (StataCorp LP, TX, and USA) and R software (version 3.5.0).

## Results

### The Microarray Analysis of Patients With T2N0M1 and T3/4N0M0

**Figure 1:**
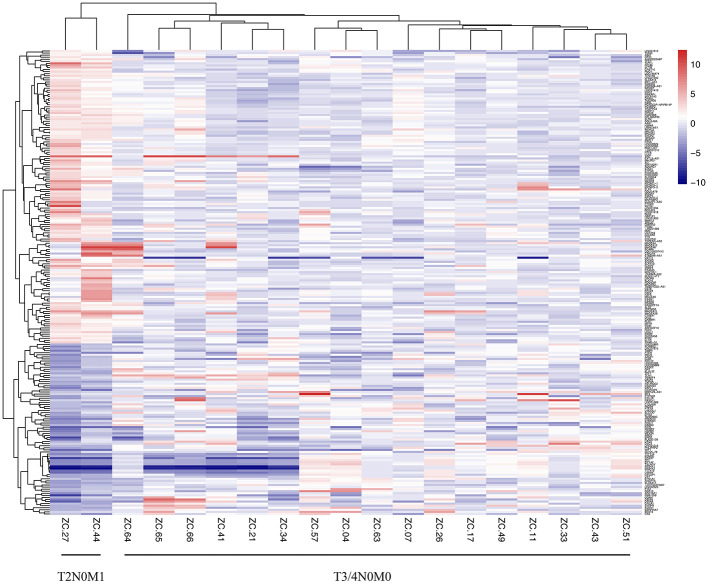
Heatmap of mRNA array of CRC patients with T2N0M1 and T3/4N0M0 samples. The mRNA profiles of the primary tumor tissues (2 samples of T2N0M1, 17 samples of T3/4N0M0) were analyzed. The heat map of mRNA array showed that there was an obvious difference between the T2N0M1 subgroup and the T3/4N0M0 subgroup.

We collected 2 CRLM patients of T2N0, who were supposed to have a low distant metastatic probability and 17 patients of T3/4N0M0 who should have a greater probability of metastasis. The detailed characteristics of 19 patients were recorded in Supplemental Table 1. The heat map of cDNA microarray showed that there was an obvious difference between the T2N0M1 subgroup and the T3/4N0M0 subgroup (Figure 1). A total of 225 differentially expressed genes were identified.

### Clinicopathological Characteristics of Metastatic CRC Patients

The cut-off date for follow up was November 2018. Of the 14,537 patients with metastatic colorectal cancer, 5,709 (39.27%) were only liver metastasis, 552 (3.88%) were only lung metastasis, 39 (0.27%) were only bone metastasis, 33 (0.23%) were only brain metastasis and 8,204 (56.44%) had multiple organic metastasis. We selected a total of 5,618 eligible liver metastatic patients into the main cohort (Selection in Supplemental Figure 1). The median follow-up time was 22 months (range, 1–83 months). The 5-year OS rate was 21.45%. Among them, 413 (7.35%) and 178 (3.17%) of patients were identified with T1 and T2 stages, while 3,424 (60.95%) and 1,603 (28.53%) were T3 and T4 stages.

### Survival Analysis

#### Survival Prognosis of Early T Stage

**Figure 2:**
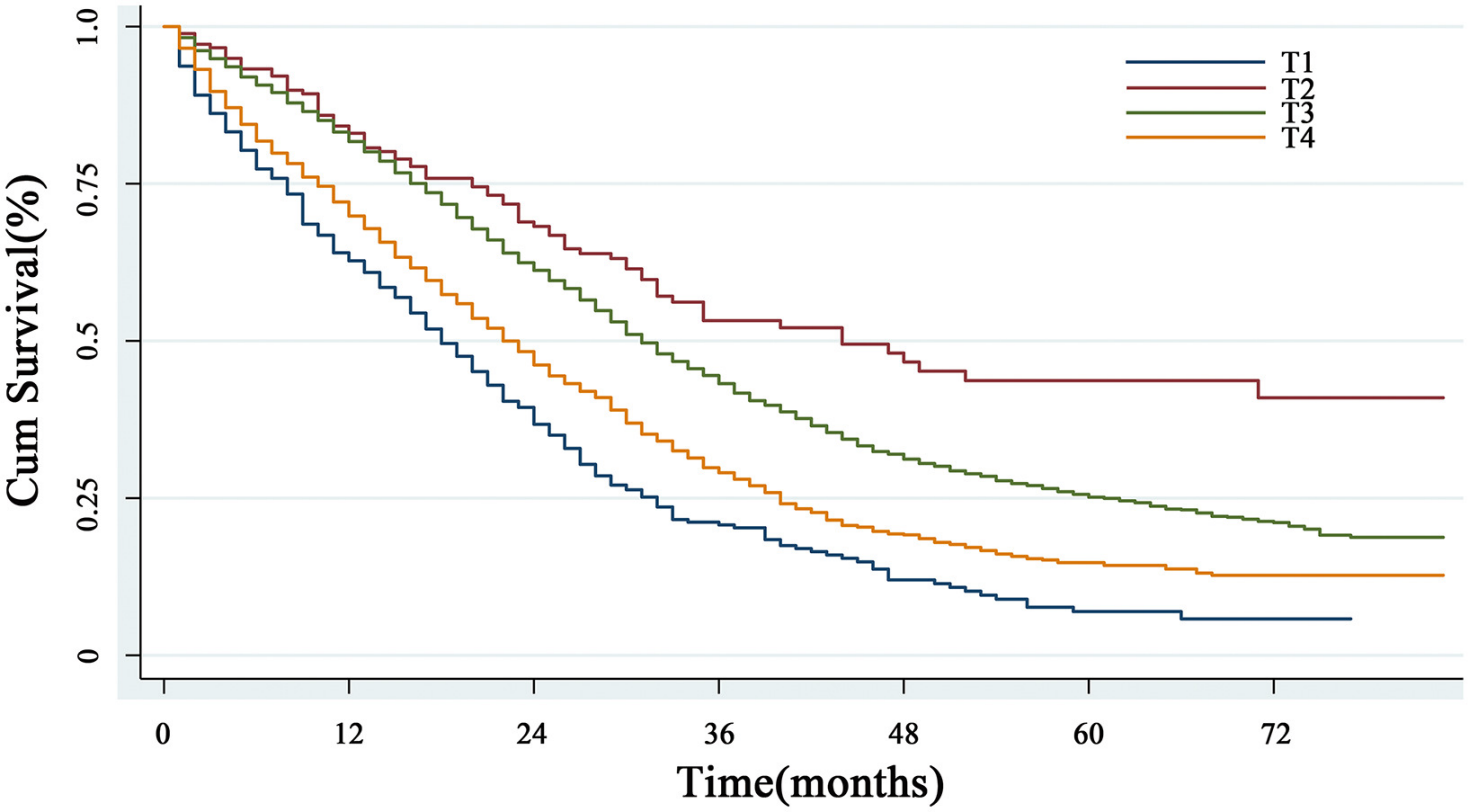
The overall survival analysis of T classification. The curves based on Kaplan-Meier method showed that patients with T1 stage had the worst prognosis (*P* < 0.001).

With respect to the impact of T staging on the CRLM patients, the univariate analysis showed that the median overall survival times were 18, 44, 31, and 22 months in the early T1, T2 cohorts and advanced T3, T4 cohorts, respectively (*P* < 0.001) (Figure 2). Surprisingly, patients with T1M1 had dramatically poor prognosis than patients with T4M1. Further multivariate Cox regression analysis including the confounding factors (age, gender, marital status, histological type, differential grade, T stage, N stage as well as CEA level), revealed that early T stage was an independent prognostic factor with poorer survival in patients with CRLM (*P* < 0.001) (Table 1).

**Table 1:** Univariate and Multivariate analysis of prognostic factors for CRLM patients.

**Variable**	**Univariate analyses**		**Multivariate analyses**	
	**HR (95%CI)**	***P*^**b**^**	**HR (95%CI)**	***P*^**b**^**
Age				
≤ 60 yrs	1		1	
>60 yrs	1.42(1.33-1.52)	0.000	1.41(1.33-1.52)	0.000
Gender				
Female	1		−	−
Male	1.01(0.94-1.08)	0.799	−	−
Race				
White	1		1	
Black	1.27(1.17-1.40)	0.000	1.26(1.16-1.38)	0.000
Other	0.89(0.79-0.99)	0.043	0.91(0.81-1.02)	0.108
Marriage				
Married	1		1	
Divorced	1.33(1.22-1.45)	0.000	1.24(1.14-1.35)	0.000
Single	1.27(1.17-1.39)	0.000	1.30(1.19-1.41)	0.000
Unknown	1.16(0.99-1.40)	0.062	1.08(0.92-1.27)	0.320
Location				
Colon	1		1	
Rectum	0.84(0.77-0.92)	0.000	0.94(0.85-1.03)	0.160
Histology				
Adenocarcinoma	1		1	
Mucinous adenocarcinoma	1.30(1.13-1.49)	0.000	1.23(1.07-1.40)	0.003
Signet ring cell carcinoma	1.27(0.68-2.37)	0.445	0.89(0.48-1.67)	0.724
Differential grade				
Well	1		1	
Moderate	0.88(0.75-1.04)	0.137	0.91(0.77-1.07)	0.234
Poor/Undifferentiated	1.42(1.20-1.68)	0.000	1.37(1.15-1.62)	0.00
T-classification^a^				
T1	1		1	
T2	0.35(0.27-0.45)	0.000	0.36(0.28-0.46)	0.000
T3	0.51(0.45-0.57)	0.000	0.45(0.39-0.51)	0.000
T4	0.76(0.67-0.86)	0.870	0.64(0.55-0.73)	0.000
N-classification^a^				
N0	1		1	
N1	0.96(0.87-1.05)	0.337	1.04(0.95-1.14)	0.402
N2	1.25(1.14-1.36)	0.000	1.32(1.20-1.45)	0.000
CEA^c^				
Negative	1		1	
Positive	1.72(1.54-1.92)	0.000	1.64(1.47-1.82)	0.000
Unknown	1.73(1.53-1.94)	0.000	1.66(1.47-1.87)	0.000

CRLM, metastatic liver colorectal cancer; CEA, carcinoembryonic antigen.

a T classification was classified as T1, T2, T3, T4 subgroups and N classification was classified as N0, N1, N2 according to the 7th AJCC TNM staging system.

b Univariate and multivariate analyses were conducted using Cox proportional hazards regression model.

c * The reference value of CEA : nonsmoker <2.5ng/ml; smoker <5ng/ml*.

#### Clinical Characteristics and Prognosis of CRLM Patients With T1/2

Patients with early T stage (T1/2) more often have tumors located in the rectum, with well differentiation, less lymph node metastasis and a higher CEA level. The detailed clinicopathological characteristics are provided in Table 2. We further explore the survival factor in patients with early T stage (T1/2M1). The multivariate analysis showed that age > 60yrs, patients without lymph node metastasis, poor differentiated tumors and a high CEA level were independently associated with poorer prognosis. There was no difference in factors of gender, race, tumor location and histology (Table 3).

**Table 2:** The characteristics of CRLM patients in early and advanced T classification.

**Risk Factors**	***N* (%)**	**T1/2 classification^**a**^** ***n* (%)**	**T3/4 classification^**a**^** ***n* (%)**	***P*^**b**^**
Total	5,618	591(10.52)	5,027(89.48)	
Age				0.628
≤ 60 yrs	2,932(52.19)	306(51.78)	2,626(52.24)	
>60 yrs	2,686(47.81)	285(48.22)	2,401(47.76)	
Gender				0.158
Female	2,281(40.60)	224(37.90)	2,057(40.92)	
Male	3,227(59.40)	367(61.10)	2,970(59.08)	
Race				0.246
White	4,198(74.72)	425(7.91)	3,733(74.05)	
Black	874(15.56)	101(17.09)	773(15.38)	
Other	546(9.72)	65(11.00)	481(9.57)	
Marriage				
Married	3,196(56.89)	321(54.31)	2,875(57.19)	0.425
Divorced	1,054(18.76)	110(18.61)	944(18.78)	
Single	1,114(19.83)	130(22.00)	984(19.57)	
Unknown	254(4.52)	38(5.88)	224(4.46)	
Location				0.000
Colon	4,650(82.77)	409(69.20)	4,650(82.77)	
Rectum	968(17.23)	182(30.80)	968(17.23)	
Histology				0.001
Adenocarcinoma	5,285(94.07)	575(97.29)	4,710(93.69)	
Mucinous adenocarcinoma	316(5.62)	14(2.37)	302(6.01)	
Signet ring cell carcinoma	17(0.30)	2(0.34)	15(0.30)	
Differential grade				0.000
Well	227(4.84)	42(7.11)	185(3.68)	
Moderate	4,131(73.53)	471(79.70)	3,660(75.44)	
Poor/Undifferentiated	1,260(22.43)	78(13.20)	1,182 (23.51)	
N-classification				0.000
N0	1,331(23.69)	349(59.05)	982(20.02)	
N1	2,252(40.09)	203(34.35)	2,049(40.76)	
N2	2,035(36.22)	39(6.60)	1,996(39.71)	
CEA ^c^				0.000
Negative	784(13.96)	59(9.98)	725(14.42)	
Positive	3,336(59.38)	394(66.67)	2,942(58.52)	
Unknown	1,498(26.66)	138(23.35)	1,360(27.05)	

CRLM, metastatic liver colorectal cancer; CEA, carcinoembryonic antigen.

a T classification was classified as T1, T2, T3, T4 subgroups and N classification was classified as N0, N1, N2 according to the 7th AJCC TNM staging system.

b P values were calculated using Chi-Squared tests.

c * The reference value of CEA : nonsmoker <2.5ng/ml; smoker <5ng/ml*.

**Table 3:** Multivariate analysis of CRLM patients with T1/2 classification.

**Variable**	**Multivariate analyses**	
	**HR (95%CI)**	***P*^**b**^**
Age		
≤ 60 yrs	1	
>60 yrs	1.38(1.12-1.70)	0.002
Gender		
Female	1	
Male	0.93(0.75-1.15)	0.501
Race		
White	1	
Black	1.29(0.98-1.70)	0.061
Other	1.04(0.75-1.45)	0.810
Marriage		
Married	1	
Divorced	1.26(0.96-1.65)	0.099
Single	1.46(1.13-1.90)	0.004
Unknown	0.99(0.63-1.54)	0.951
Location		
Colon	1	
Rectum	0.99(0.79-1.25)	0.968
Histology		
Adenocarcinoma	1	
Mucinous adenocarcinoma	1.46(0.79-2.71)	0.226
Signet ring cell carcinoma	3.63(0.89-14.80)	0.073
Differential grade		
Well	1	
Moderate	0.88(0.59-1.30)	0.511
Poor/Undifferentiated	1.61(1.03-2.52)	0.038
N-classification^a^		
N0	1	
N1	0.72(0.62-0.91)	0.005
N2	0.49(0.30-0.79)	0.004
CEA^c^		
Negative	1	
Positive	2.28(1.47-3.54)	0.000
Unknown	2.09(1.31-3.34)	0.002

CRLM, metastatic liver colorectal cancer; CEA, carcinoembryonic antigen.

a N classification was classified as N0, N1, N2 according to the 7th AJCC TNM staging system.

b P values was conducted using Cox proportional hazards regression model.

c *The reference value of CEA : nonsmoker <2.5ng/ml; smoker <5ng/ml*.

#### Survival Influence of N Status on CRLM Patients of T1/2

**Figure 3:**
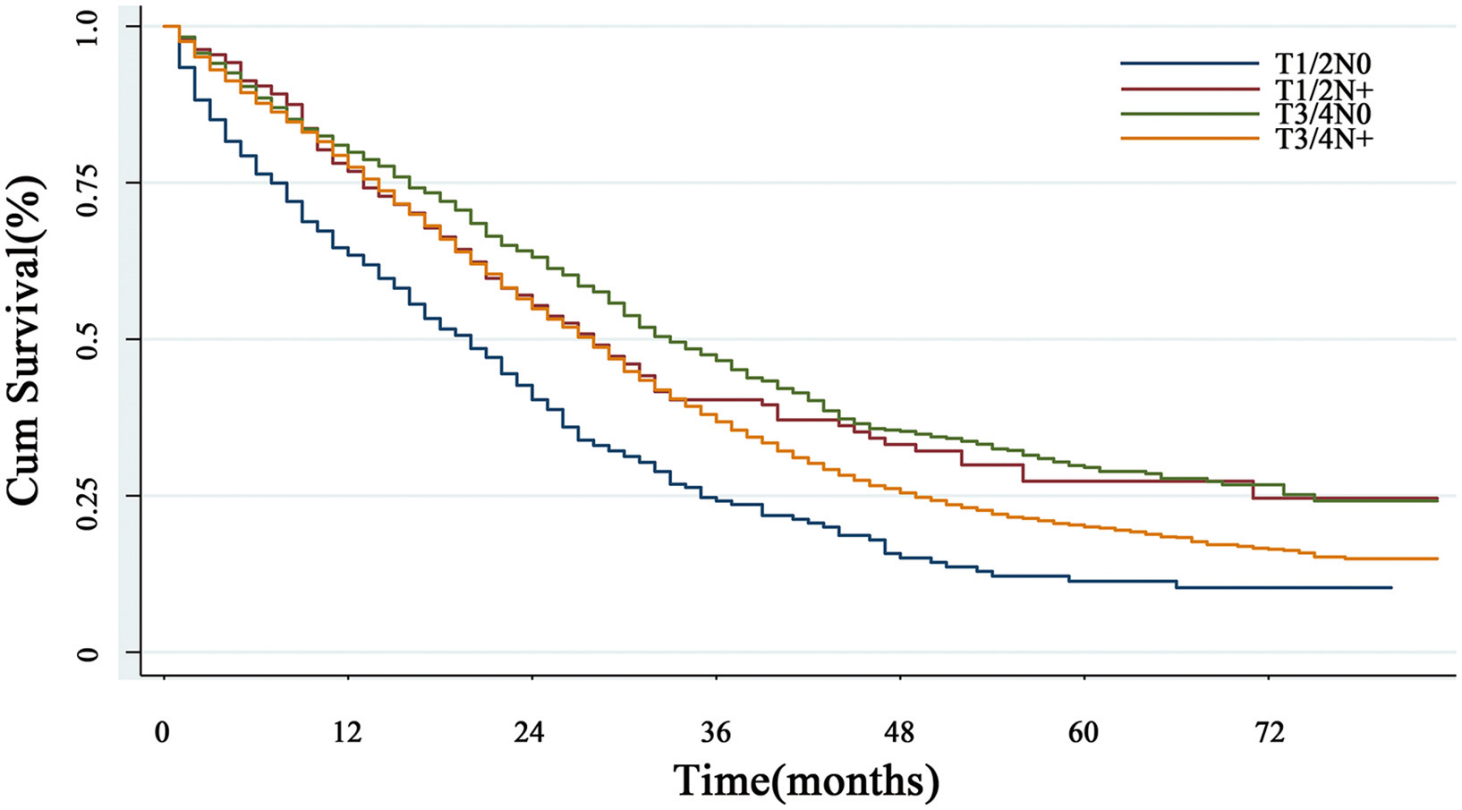
The overall survival in subgroup analysis with lymph node status. When the N status was taken into consideration, the survival curve showed that T1/2N+ subgroup had the worst prognosis, while the prognosis of T3/4N0 was the best (*P* < 0.001).

Lymph node status is also a vital factor which should also be taken into consideration. Due to the limited sample size, we divided the cohort into N0 (lymph node negative) and N+ (lymph node positive) subgroups. When the N status was taken into consideration, the survival curve surprisingly showed that T1/2N+ subgroup had the worst prognosis, while the prognosis of T3/4N0 was the best (*P* < 0.001) (Figure 3).

### Construction and Validation of a Prognostic Risk Nomogram

**Figure 4:**
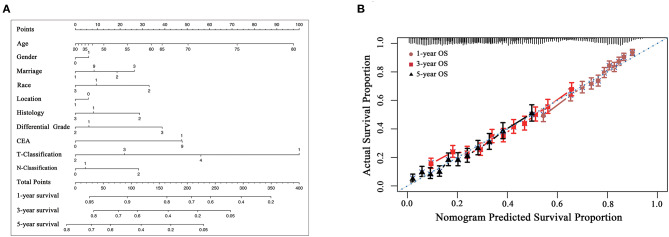
**(A)** preoperative prognostic nomogram model for patients with CRLM. (To use the nomogram model, an individual patient's value is located on each variable axis, and a line is drawn upward to determine the number of points received for each variable value. The sum of these numbers is located on the Total Points axis, and a line is drawn downward to the survival axes to determine the likelihood of 1−, 3− or 5-year overall survival). Gender: 0, male, 1, female; Marriage: 1, married, 2, divorced, 3, single, 9, unknown; Race: 1, white, 2, black, 3, others; Location: 0, colon, 1, rectum; Histology: 1, adenocarcinoma, 2, mucinous adenocarcinoma, 3, signet ring cell carcinoma; Grade: 1, well differentiated, 2, moderately differentiated, 3, poorly differentiated/undifferentiated, 4, undifferentiated; CEA: 0, negative, 1, positive, 9, unknown. CEA, carcinoembryonic antigen. The reference value of CEA: nonsmoker <2.5 ng/ml; smoker <5 ng/ml. **(B)** The calibration curve for predicting patient survival at 1−, 3−, and 5− years. Nomogram-predicted probability of overall survival is plotted on the *x*-axis; actual overall survival is plotted on the *y*-axis [C-index of 0.652 (95%CI 0.642-0.662)].

A nomogram model based on prognostic preoperative factors, including age, gender, marital status, race, tumor location, histological type, grade, CEA status, T/N stage were established (Figure 4A). The nomogram facilitates the easy and simultaneous consideration of prognostic factors. As shown in the nomogram, T stage had the largest contribution to prognosis, with CEA and histological type following. Each subtype within these variables was assigned a score on the point scale (Table 4). By adding up the total score and locating it on the total point scale, we were able to draw a straight line down to provide estimates of 1−, 3− or 5− year predicted OS.

**Table 4:** Point assignment and prognostic score in nomogram.

**Variables**	**Score**	**Estimated 3-year CIF (%)**
Age		
20-24	0	
25-29	1	
30-34	3	
35-39	4	
40-44	6	
45-49	8	
50-54	13	
55-59	23	
60-64	34	
65-69	39	
70-74	50	
75-79	72	
>=80	98	
Gender		
Female	0	
Male	6	
Race		
White	0	
Black	3	
Other	0	
Marriage		
Married	0	
Divorced	19	
Single	26	
Unknown	8	
Location		
Colon	6	
Rectum	0	
Histology		
Adenocarcinoma	8	
Mucinous adenocarcinoma	29	
Signet ring cell carcinoma	0	
Differential grade		
Well	6	
Moderate	0	
Poor/Undifferentiated	39	
T classification^a^		
T1	100	
T2	0	
T3	1	
T4	4	
N classification^a^		
N0	0	
N1	0	
N2	2	
CEA		
Negative	0	
Positive	48	
Unknown	48	
Total prognostic score (3-year CIF)		
277		0.05
219		0.20
166		0.40
111		0.60
77		0.70
33		0.80

CIF, Cumulative Incidence Function.

a *T classification was classified as T1, T2, T3, T4 subgroups and N classification was classified as N0, N1, N2 according to the 7th AJCC TNM staging system*.

The model was internally validated using the bootstrap validation method. The model demonstrated acceptable accuracy for predicting OS, with a C-index of 0.652 (95%CI 0.642-0.662). Calibration curves for 1−, 3−, and 5-year OS estimates revealed acceptable model calibration, with good correlation between the OS estimates from the nomogram model and those derived from Kaplan-Meier estimates (Figure 4B).

## Discussion

Our study compared the patients at T2N0M1, who were assumed to have a low distant metastatic propensity, and the patients at T3/4N0M0, which should have a greater probability of metastasis by using the cDNA microarray to explore the potential biological differences. Results indicated that metastatic colorectal patients at early T stage (T1/2) had a unique genetic profile. Those differentially expressed genes might be potential indicators of patients with poor prognoses, as well as therapeutic targets.

Furthermore, our study analyzed the clinical and survival characteristics of CRLM patients from the SEER database. The results illustrated that the prognosis of patients at T1 stage was extremely unfavorable. More surprisingly, patients without lymph node metastasis even had a dismal outcome. Most previous studies (11, 12) indicated that patients with advanced T stage (T3/4) had significantly bad prognoses. In order to predict the likelihood of tumor recurrence and the survival after the resection of CRLM, a panel of Clinical Risk Scores (CRS) have been assessed. The most widely used and validated CRS has been described by Fong et al. in 1999 (13). Its prognostic value has been confirmed by several independent research groups (14–16). Among the 5 criteria, the N status was highlighted, albeit without mentioning the T stage. However, our present study underscored that the influence of T staging might be largely underestimated. Although the CRS could estimate partially ‘high risk' individuals, an undefined group which was previously regarded as ‘low risk' might be authentically high risk.

There is no previous research mentioned the above “odd” T1 stage in CRLM, but some studies revealed a similar phenomenon. Recently, a clinical case (17) reported a type of “extra-luminal” recurrence occurred in a patient with a low-risk T1 colorectal carcinoma. The patients initially presented with a 15-mm sessile polyp located in the rectum which was diagnosed as a low-risk case of T1 colorectal cancer. During the follow-up period, a 15-mm rectal adenocarcinoma reoccurred, extending from the muscularis propria to the perirectal adipose tissue. Moreover, a Japanese study (18) reported that there were some cases in T1 CRCs with “skip lymphovascular invasion” (SLVI), which is defined as the discontinuous foci of the tumor cells within the colonic layers. The study suggested that skip lymphovascular invasion was associated with hepatic metastasis in CRC cases. Yuta sato et al. (19) have analyzed the clinicopathological features of T1 CRC with SLVI and indicated that lymphovascular invasion was a significant risk factor for SLVI in T1 CRC. The above three studies consistently implicated the possible existence of an undefined group of T1 CRC.

This phenomenon of poor T1M1 which has not been reported in previous studies might be attributable to the following reasons. First, patients at T1/2M1 might be too peculiar to be collected for quantitative analyses. This might be causative to explaining no characterizations in previous analyses of the SEER database. Second, the treatment of mCRC has become largely effective since Oxaliplatin has been marketed in 2001 and widely used in clinic. The survival of most patients with mCRC has been significantly improved, leading to the emergence of a specific group of T1M1 patients with extremely poor prognosis.

Nowadays, modern methods of endoscopic treatment have been fast developed, including endoscopic mucosal resection (EMR) and endoscopic submucosal dissection (ESD). An increasing number of T1 CRCs are resected endoscopically, which underlines an urgent focus on these poor T1M1 patients. In our study, we developed and validated a nomogram model for preoperative individualized prediction which can directly predict the long-term possibility of survival for patients. Incorporating the preoperative clinical factors into an easy-to-use nomogram facilitates clinicians to evaluate the patients. The definition of “high risk” is based on the total risk score, providing an unbiased scaling system to evaluate patients and to further propel the clinical decision making. Our preoperative nomogram model revealed that early T staging played a significant role. It possibly even matters to escalate adequate treatment approaches for metastatic patients at early T stage. Consequently, since the detection of patients with T1M1 at an early time became important, we might need to rethink the treatment of early stage colorectal cancer. Regarding the existence of a 10% (10) deadly rate in early colorectal patients, it is tempting to point out that there might be a special group of patients with poor T1 mixed at the early stage. When analyzing the non-metastatic CRC patients, we found that the poor T1 stage in M0 group could not be obtained (data not shown). This is proportionally because a large number of T1/2 patients with good prognoses overlapped or even counteracted the poor effect from some patients. Even including some other digestive tumors such as gastric cancer and esophageal cancer as sensitive analyses, no similar result of T stage was found (data not shown). For these poor patients with T1 stage CRLM, it is rational to initially select the specific high-risk patients based on relevant clinical risk parameters in combination with other possible biological markers provided by tissue microarray and second-generation sequencing and to characterize them with more clinical attention. Indeed, those T1M1 patients will pose a significant challenge and might not be the suitable candidates for immediate metastasectomy. Instead, adjuvant therapy may be the priority, which makes surgery more effective.

Our present study also has some potential limitations. First, about the cDNA microarray, there is only two patients with T1/2M1 stage, and no experiments were performed to explore the potential roles of the different genes. More researches will be performed in subsequent experiments. Second, it was a retrospective study. As a population-based registration database, the SEER database has inevitable inaccuracy. Third, several popular variables including the AFP levels are not available in the SEER database. Information about the disease-free survival and details about pre- or post-operative therapies including the chemotherapeutic agents are also lacking. Nevertheless, the above issues are often unavoidable in survival studies. Therefore, we focus on the poor prognosis of T1 patients in patients with CRLM. CRLM with early T stage had a paradoxical biological background. The prognosis of T1/2M1 patients was poorer than that of T3/4M1. The study critically discerned a rare but vicious subtype in CRC. More endeavors should be made toward the high-risk surveillance and identification of patients at early T stage. Stage IV patients might further necessitate their characterizations of T staging.

## Data Availability Statement

The original contributions presented in the study are publicly available. This data can be found here: the NCBI Gene Expression Omnibus (GSE146480).

## Ethics Statement

The studies involving human participants were reviewed and approved by ethics committee of Second Affiliated Hospital of Zhejiang University School of Medicine. The patients/participants provided their written informed consent to participate in this study.

## Author Contributions

SZ and LWa: conception and design. LWu and JF: collection, analysis, and interpretation of data. All authors: manuscript writing and editing and final approval of manuscript.

## Conflict of Interest

The authors declare that the research was conducted in the absence of any commercial or financial relationships that could be construed as a potential conflict of interest.

## Supplementary Material

Supplementary Figure 1 Cohort inclusion and exclusion diagram.

## Abbreviations

SEER: Surveillance, Epidemiology and End Results
CRC: Colorectal cancer
mCRC: Metastatic colorectal cancer
CRLM: Metastatic liver colorectal cancer
OS: Overall survival
CEA: Carcinoembryonic antigen
HRs: Hazard ratios
CI: Confidence intervals
CEA: Carcinoembryonic antigen
NOS: None of specific
AJCC: American Joint Committee on Cancer.
