# Supplementary figures and images for: Early T Stage Is Associated With Poor Prognosis in Patients With Metastatic Liver Colorectal Cancer
Source: Front Oncol. 2020 Jun 18;10:716. doi: 10.3389/fonc.2020.00716 (PMC7314979; doi:10.3389/fonc.2020.00716)

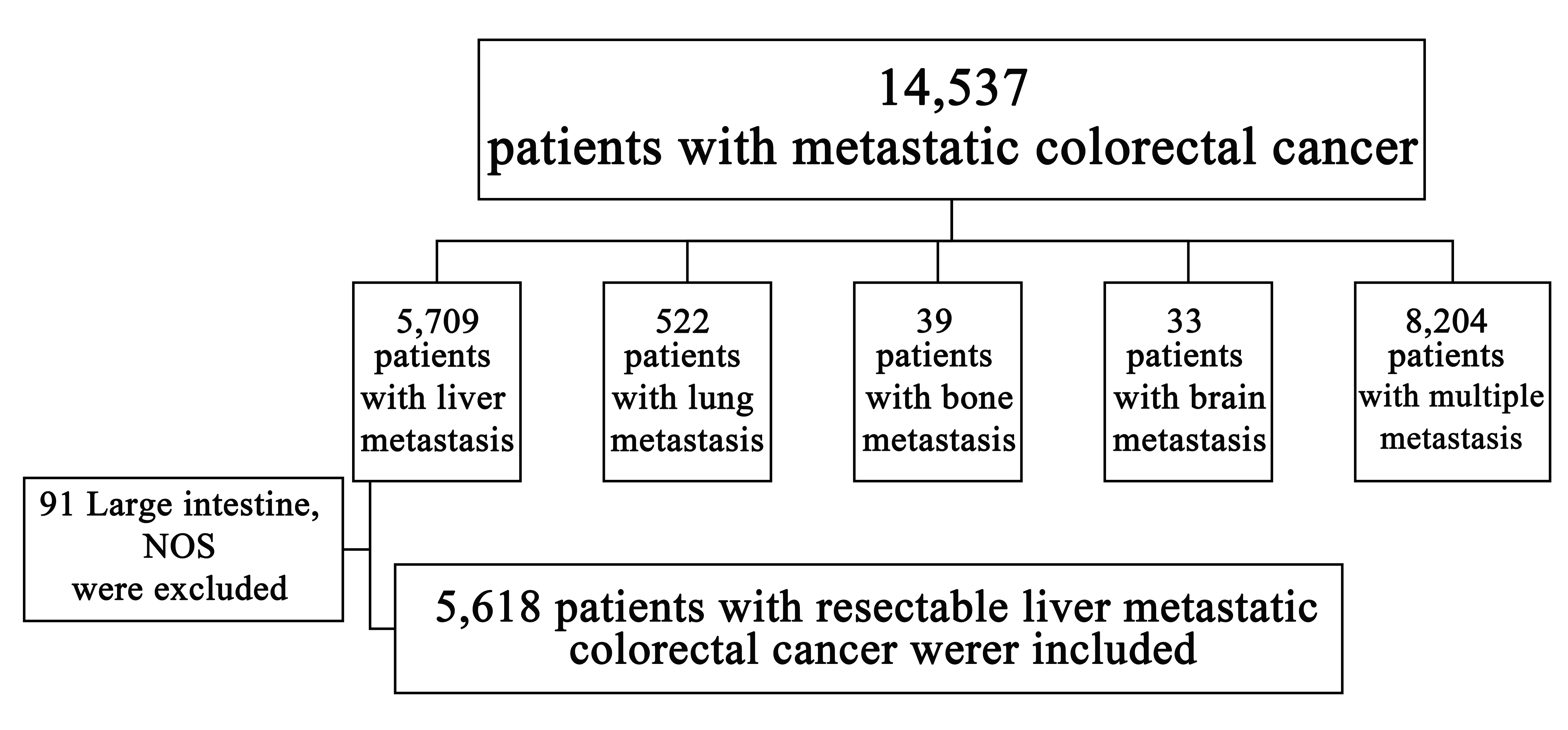

Supplement: Supplementary Figure 1 — Cohort inclusion and exclusion diagram. [file Image_1.TIF]
