# Supplementary material for: Early T Stage Is Associated With Poor Prognosis in Patients With Metastatic Liver Colorectal Cancer
Source: Front Oncol. 2020 Jun 18;10:716. doi: 10.3389/fonc.2020.00716 (PMC7314979; doi:10.3389/fonc.2020.00716)
Supplement: Supplementary file 2 [file Table_1.DOCX]

Supplemental Table 1. Characteristics of 19 patients for mRNA profile analysis

|  | Patient ID | Location | Gender | Age | Stage | TNM |
| --- | --- | --- | --- | --- | --- | --- |
| T3/4M0 | zc64 | Rectum | Male | 43 | II | T4N0M0 |
|  | zc51 | Rectum | Male | 48 | II | T4N0M0 |
|  | zc11 | Colon | Male | 56 | II | T3N0M0 |
|  | zc7 | Colon | Male | 55 | II | T4N0M0 |
|  | zc34 | Colon | Female | 61 | II | T3N0M0 |
|  | zc66 | Rectum | Female | 53 | II | T3N0M0 |
|  | zc33 | Rectum | Male | 61 | II | T3N0M0 |
|  | zc26 | Colon | Male | 67 | II | T4N0M0 |
|  | zc21 | Colon | Female | 71 | II | T3N0M0 |
|  | zc57 | Colon | Male | 74 | II | T4N0M0 |
|  | zc4 | Rectum | Male | 40 | II | T3N0M0 |
|  | zc17 | Rectum | Male | 47 | II | T3N0M0 |
|  | zc63 | Rectum | Male | 53 | II | T3N0M0 |
|  | zc65 | Colon | Female | 52 | II | T3N0M0 |
|  | zc41 | Colon | Female | 47 | II | T4N0M0 |
|  | zc49 | Rectum | Male | 57 | II | T4N0M0 |
|  | zc43 | Colon | Male | 61 | II | T3N0M0 |
| T2M1 | zc44 | Rectum | Female | 57 | IV | T2N0M1 |
|  | zc27 | Colon | Female | 58 | IV | T2N0M1 |
|  |  | | | | | |
